# Activin E is a new guardian protecting against hepatic steatosis via inhibiting lipolysis in white adipose tissue

**DOI:** 10.1038/s12276-025-01403-6

**Published:** 2025-02-13

**Authors:** Shi-Young Park, Yoonil Cho, Sae-Mi Son, Jang Ho Hur, Yeongmin Kim, Hyunhee Oh, Hui-Young Lee, Sungwon Jung, Sanghee Park, Il-Young Kim, Se-Jin Lee, Cheol Soo Choi

**Affiliations:** 1 Korea Mouse Metabolic Phenotyping Center, Lee Gil Ya Cancer and Diabetes Institute, Gachon University, Incheon, Republic of Korea; 2 Gachon Biomedical Convergence Institute, Gachon University Gil Medical Center, Incheon, Republic of Korea; 3 Department of Health Sciences and Technology, GAIHST, Gachon University, Incheon, Republic of Korea; 4 Integrative Metabolic Fluxomics Lab, Lee Gil Ya Cancer and Diabetes Institute, Gachon University, Incheon, Republic of Korea; 5 Division of Molecular Medicine, Department of Medicine, Gachon University College of Medicine, Incheon, Republic of Korea; 6 Department of Genome Medicine and Science, Gachon University College of Medicine, Incheon, Republic of Korea; 7 Gachon Institute of Genome Medicine and Science, Gachon University Gil Medical Center, Incheon, Republic of Korea; 8 Department of Exercise Rehabilitation, Gachon University, Incheon, Republic of Korea; 9 The Jackson Laboratory for Genomic Medicine, Farmington, CT USA; 10 Department of Genetics and Genome Sciences, University of Connecticut School of Medicine, Farmington, CT USA; 11 Endocrinology, Internal Medicine, Gachon University Gil Medical Center, Incheon, Republic of Korea

**Keywords:** Fat metabolism, Metabolic syndrome, Metabolic syndrome, Metabolic syndrome

## Abstract

Hepatic endoplasmic reticulum (ER) stress is implicated in the development of steatosis and its progression to nonalcoholic steatohepatitis (NASH). The ER in the liver can sustain metabolic function by activating defense mechanisms that delay or prevent the progression of nonalcoholic fatty liver disease (NAFLD). However, the precise mechanisms by which the ER stress response protects against NAFLD remain largely unknown. Recently, activin E has been linked to metabolic diseases such as insulin resistance and NAFLD. However, the physiological conditions and regulatory mechanisms driving hepatic *Inhbe* expression (which encodes activin E) as well as the metabolic role of activin E in NAFLD require further investigation. Here we found that hepatic *Inhbe* expression increased under prolonged fasting and ER stress conditions, which was mediated by ATF4, as determined by promoter analysis in a mouse model. Consistently, a positive correlation between *INHBE* and *ATF4* expression levels in relation to NAFLD status was confirmed using public human NAFLD datasets. To investigate the role of activin E in hepatic steatosis, we assessed the fluxes of the lipid metabolism in an *Inhbe*-knockout mouse model. These mice displayed a lean phenotype but developed severe hepatic steatosis under a high-fat diet. The deficiency of *Inhbe* resulted in increased lipolysis in adipose tissue, leading to increased fatty acid influx into the liver. Conversely, hepatic overexpression of *Inhbe* ameliorated hepatic steatosis by suppressing lipolysis in adipose tissue through ALK7–Smad signaling. In conclusion, activin E serves as a regulatory hepatokine that prevents fatty acid influx into the liver, thereby protecting against NAFLD.

## Activin E protects against fatty liver by inhibiting fat breakdown

Hepatic endoplasmic reticulum (ER) stress is associated with nonalcoholic fatty liver disease (NAFLD). Although it is known that the liver’s ER can activate defense mechanisms to slow NAFLD progression, the specific protective processes remain unclear. Recent studies have identified activin E, encoded by the *Inhbe* gene, as being linked to metabolic conditions such as insulin resistance and NAFLD. This research demonstrates that hepatic *Inhbe* expression increases during ER stress, a process regulated by ATF4. Loss of *Inhbe* led to severe hepatic steatosis due to enhanced lipolysis in adipose tissue. By contrast, hepatic overexpression of *Inhbe* alleviated hepatic steatosis by suppressing adipose tissue lipolysis through ALK7–Smad signaling. These findings suggest that activin E functions as a regulatory hepatokine, protecting against NAFLD by preventing excessive fatty acid influx into the liver through the inhibition of adipose lipolysis.

This summary was initially drafted using artificial intelligence, then revised and fact-checked by the author.

## Introduction

Nonalcoholic fatty liver disease (NAFLD) is a common chronic liver disease with an estimated global prevalence of 32.4%^1^. Endoplasmic reticulum (ER) stress has been implicated in the development of steatosis and its progression to nonalcoholic steatohepatitis (NASH)^2–4^. Various chronic disturbances, including lipotoxicity, can induce ER stress, leading to dysregulated hepatic lipid metabolism, insulin resistance and inflammation, which are key contributors to the pathogenesis of NAFLD^5^. Conversely, during disease progression, the hepatic ER can activate defense mechanisms to maintain metabolic function and delay or prevent the progression of NAFLD^6^. However, the precise mechanisms through which ER stress protects against NAFLD remain largely unknown.

Activins, which are members of the TGF-β superfamily, are implicated in various biological processes^7^. There are four main types of activin: activins A, B, C and E, which are encoded by *Inhba*, *Inhbb*, *Inhbc* and *Inhbe*, respectively. Activins A and B play physiological roles in regulating glucose and energy metabolism by modulating the functions of the pancreas, liver, muscles and adipose tissues^8,9^. Under pathological conditions, serum activin A levels are positively correlated with NAFLD^10^. Activin B has been identified as a potential adipokine that regulates glucose and energy metabolism and is positively associated with obesity^11^. However, the roles of activins C and E remain unknown and have not been extensively studied.

Recent studies have suggested that activin E, which is predominantly expressed in the liver, is positively associated with insulin resistance in human liver biopsy samples^12^, and drug-induced ER stress upregulates the expression of *Inhbe*^13^, suggesting its potential involvement in the development of NAFLD. However, the physiological and pathological conditions responsible for *Inhbe* expression and the regulatory mechanisms of *Inhbe* expression in the liver remain unclear. Moreover, the metabolic role of activin E in ER stress during NAFLD requires further investigation.

In this study, to elucidate the role of activin E in NAFLD, we first analyzed the correlation between the expression of *INHBE* and NAFLD status using public datasets of NAFLD in humans. In addition, we investigated the mechanisms of *Inhbe* expression under hepatic ER stress. We also aimed to elucidate the mechanisms underlying the development of hepatic steatosis by measuring the fluxes of various lipid metabolic pathways in an *Inhbe*-knockout (KO) mouse model. Finally, we demonstrated that activin E plays a central role in the regulation of adipose tissue lipolysis. Our findings strongly indicate that activin E acts as a regulatory hepatokine by preventing the influx of fatty acids from adipose tissue into the liver, thereby protecting against NAFLD under ER stress.

## Materials and methods

### Clinical relevance analysis of *INHBE*

We analyzed the clinical relevance of human *INHBE* expression using five transcriptome datasets from the Gene Expression Omnibus (GEO) and European Nucleotide Archive public databases.

### Animal study

C57BL/6J mice (males, 8 weeks old) were purchased from Japan SLC, and *Inhbe*-KO mice were generated by Dr. Se-Jin Lee. All the animal studies were performed in accordance with the regulations and guidelines of the Institutional Animal Care and Use Committee of the Center of Animal Care and Use at the Lee Gil Ya Cancer and Diabetes Institute, Gachon University.

### Hyperinsulinemic–euglycemic clamp study with the isotope dilution method

After an overnight fast, [3-^3^H]-glucose (HPLC purified; PerkinElmer) was infused at a rate of 0.05 μCi/min for 2 h to assess basal glucose turnover. Following this basal period, a hyperinsulinemic–euglycemic clamp was conducted for 120 min with a primed/continuous infusion of human insulin (21.4 mU/kg during priming and 3 mU/kg/min during infusion, Eli Lilly), while plasma glucose was maintained at basal concentrations, as previously described, with slight modifications^14^. To estimate insulin-stimulated whole-body glucose flux, [3-^3^H]-glucose was infused at a rate of 0.1 μCi/min throughout the clamp. Details on the determination of the basal and insulin-stimulated whole-body glucose fluxes can be found in the Supplementary Information.

### Stable isotope metabolic flux study

A stable isotope tracer infusion study was performed using infusions of [U-^13^C_16_]palmitate (#CLM-3943) and [1,1,2,3,3-D_5_]glycerol isotope tracers (#DLM-1129) (Cambridge Isotope Laboratories). After a 6-h fasting period, the mice were infused with the tracers at specific rates (palmitate at 1.85 nM/g/min and glycerol at 2.5 nM/g/min) for 120 min. Blood samples were collected at 90, 100 and 110 min to determine the plasma enrichment of both tracers. A D_2_O labeling study was performed in which the mice were administered an intraperitoneal injection of 35 μl/g body weight of 99% D_2_O (DLM-4, Cambridge Isotope Laboratories) mixed with 0.9% NaCl. The mice had ad libitum access to drinking water enriched with 8% semi-heavy water for the final 5 days. The isotopic enrichment measurements and metabolite kinetics calculations were performed by Myocare and are detailed in the Supplementary Information.

### Statistical analysis

All the data are expressed as the mean ± standard error of the mean (s.e.m.). The statistical data included results from at least three independent experiments. Statistical significance between two groups was compared using the Student’s *t*-test, and statistical significance among three groups was compared by one-way analysis of variance with post hoc analysis using GraphPad Prism software ver. 10 (GraphPad Software). A *P* value <0.05 was considered to indicate statistical significance.

The other detailed methods are presented in the Supplementary Information.

## Results

### *Inhbe* expression is increased by hepatic ER stress under physiological or pathological conditions

**Fig. 1:**
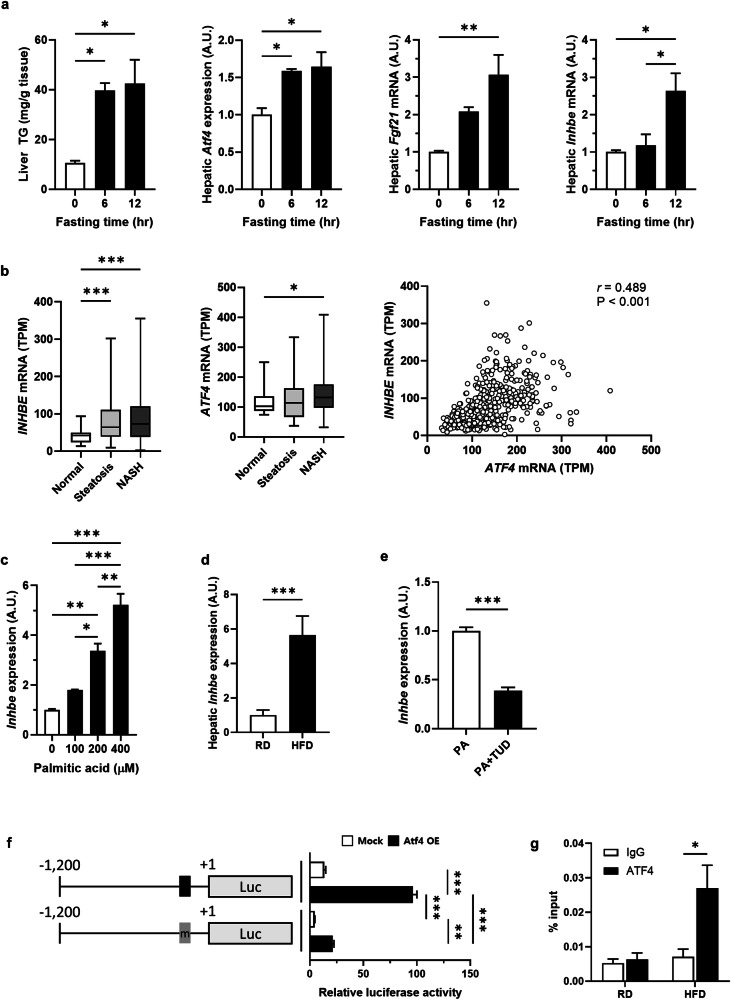
*Inhbe* expression is increased by hepatic ER stress under physiological or pathological conditions. **a** Liver TG contents and hepatic mRNA expression of *Atf4*, *Fgf21* and *Inhbe*. Twelve-week-old male C57BL/6J mice were housed under ad libitum, fasted for 6 h or fasted for 12 h (each group, *n* = 3). **b** mRNA levels of *INHBE* and *ATF4* in public datasets of RNA-sequencing data from the livers of patients with NAFLD (normal, *n* = 39; steatosis, *n* = 179; NASH, *n* = 260). Gene expression correlation analysis of *INHBE* and *ATF4* in all samples from the datasets (normal + steatosis + NASH, *n* = 454). **c** mRNA and protein levels of *Inhbe* in AML12 hepatocytes treated with 0.4 mM palmitate for 6 h at the indicated concentrations. **d** mRNA levels of *Inhbe* in the livers of mice fed a HFD for 4 weeks. **e** mRNA levels of *Inhbe* in AML12 cells treated with 0.4 mM palmitate with or without 2 mM tauroursodeoxycholic acid for 6 h. **f** Luciferase activity in AML12 cells transfected with the 1.2 kb *Inhbe* WT promoter or the putative ATF4-binding element-deleted promoter under ATF4 overexpression conditions. **g** Chromatin immunoprecipitation assay with a specific anti-ATF4 antibody and primers for the putative ATF4-binding element in the *Inhbe* promoter. The data are presented as the mean ± s.e.m. *P < 0.05, **P < 0.01, ***P < 0.001.

During prolonged fasting, the liver is exposed to excess fat due to increased lipolysis in adipose tissue, leading to fat accumulation that may induce ER stress^15^. In response to prolonged fasting, the liver activates various compensatory functions to maintain homeostasis against ER stress, such as reducing fat accumulation and ER stress responses. Therefore, we investigated the expression patterns of *Inhbe* during fasting to understand its physiological roles. First, we tested whether hepatic steatosis increased during fasting in a mouse model. As the fasting period increased, the plasma levels of free fatty acids and free glycerol increased (Supplementary Fig. 1), accompanied by a simultaneous increase in hepatic triacylglycerol (TG) levels (Fig. 1a). Consistent with previous reports on hepatic ER stress responses^16^, the expression of *Atf4* and *Fgf21* in the liver gradually increased during fasting (Fig. 1a). *Inhbe* expression was significantly elevated during late fasting. These data indicate that hepatic *Inhbe* expression is increased during prolonged fasting and that its expression is associated with ER stress.

To further confirm the clinical significance of *INHBE*, we investigated *INHBE* expression using five large-scale liver transcriptome datasets from patients with various NAFLD statuses obtained from the Gene Expression Omnibus (GEO) and European Nucleotide Archive (ENA) databases. During the progression of normal liver to hepatic steatosis or NASH, there was a significant increase in *INHBE* expression (Fig. 1b). Among the potential transcription factors activated by the fatty acids accumulated in the liver, the expression of *ATF4*, the main transcriptional regulator of ER stress, was significantly elevated in the NASH group compared with the healthy group. The expression patterns of *INHBE* and *ATF4* were significantly positively correlated (Pearson correlation coefficient (*r*) = 0.489, *P* < 0.0001) (Fig. 1b). Other transcription factors showed mild or negative correlations with the *INHBE* expression pattern (Supplementary Fig. 2a–f). Therefore, to determine whether ER stress conditions are suitable for the induction of *Inhbe* expression, we investigated *Inhbe* expression in ER stress models. Treatment of AML12 cells with palmitic acid markedly increased *Inhbe* mRNA levels (Fig. 1c) and *Atf4* expression (Supplementary Fig. 3a) in a dose-dependent manner. Chemically induced ER stress also induced the expression of *Inhbe* (Supplementary Fig. 3b,c). In the liver tissue of mice fed a high-fat diet (HFD), there was a profound increase in *Inhbe* and *Atf4* expression (Fig. 1d and Supplementary Fig. 3d), accompanied by elevated plasma levels of activin E (Supplementary Fig. 3e). Furthermore, inhibitors of ER stress, tauroursodeoxycholic acid or 4-phenyl butyric acid, suppressed *Inhbe* expression induced by palmitic acid in AML12 cells and the liver tissue of HFD-fed mice (Fig. 1e and Supplementary Fig. 3f). These data strongly suggest that hepatic ER stress induces *Inhbe* expression.

Next, we focused on the mechanism regulating hepatic *Inhbe* expression under ER stress. On the basis of our observation of a positive correlation between *Inhbe* and *Atf4* expression under NAFLD conditions, we examined whether ATF4 directly induced *Inhbe* expression via ATF4 overexpression and knockdown systems in vitro. The overexpression of *Atf4* increased *Inhbe* expression, and the knockdown of *Atf4* diminished palmitic acid-induced *Inhbe* expression in AML12 cells (Supplementary Fig. 4a,b). To define a precise regulatory mechanism, we searched for a *cis*-element sequence regulated by ATF4 in the *Inhbe* promoter using the virtual promoter analysis tool, PROMO^17^. The putative ATF4 binding sequence, TGACGTAAG, was identified at −278 to −271 base pairs (bp) upstream from the transcription initiation site compared with the ATF4 consensus sequence, TGACGXAAX (Supplementary Fig. 4c). To ascertain whether this element of the *Inhbe* promoter is crucial for ATF4-induced *Inhbe* transcription, luciferase reporter vector constructs containing 1.2 kb of the mouse *Inhbe* promoter were used. *Atf4* overexpression increased luciferase activity, but luciferase activity was diminished in the mutant vector without the putative ATF4 binding element (Fig. 1f and Supplementary Fig. 4d). In addition, we confirmed the direct binding of ATF4 to the putative element of the *Inhbe* promoter using chromatin immunoprecipitation both in vivo and in vitro (Fig. 1g and Supplementary Fig. 4e). These data clearly indicate that ATF4 is a crucial transcription factor for *Inhbe* expression under ER stress.

### The *Inhbe*-KO mouse has a lean but lipodystrophic phenotype

**Fig. 2:**
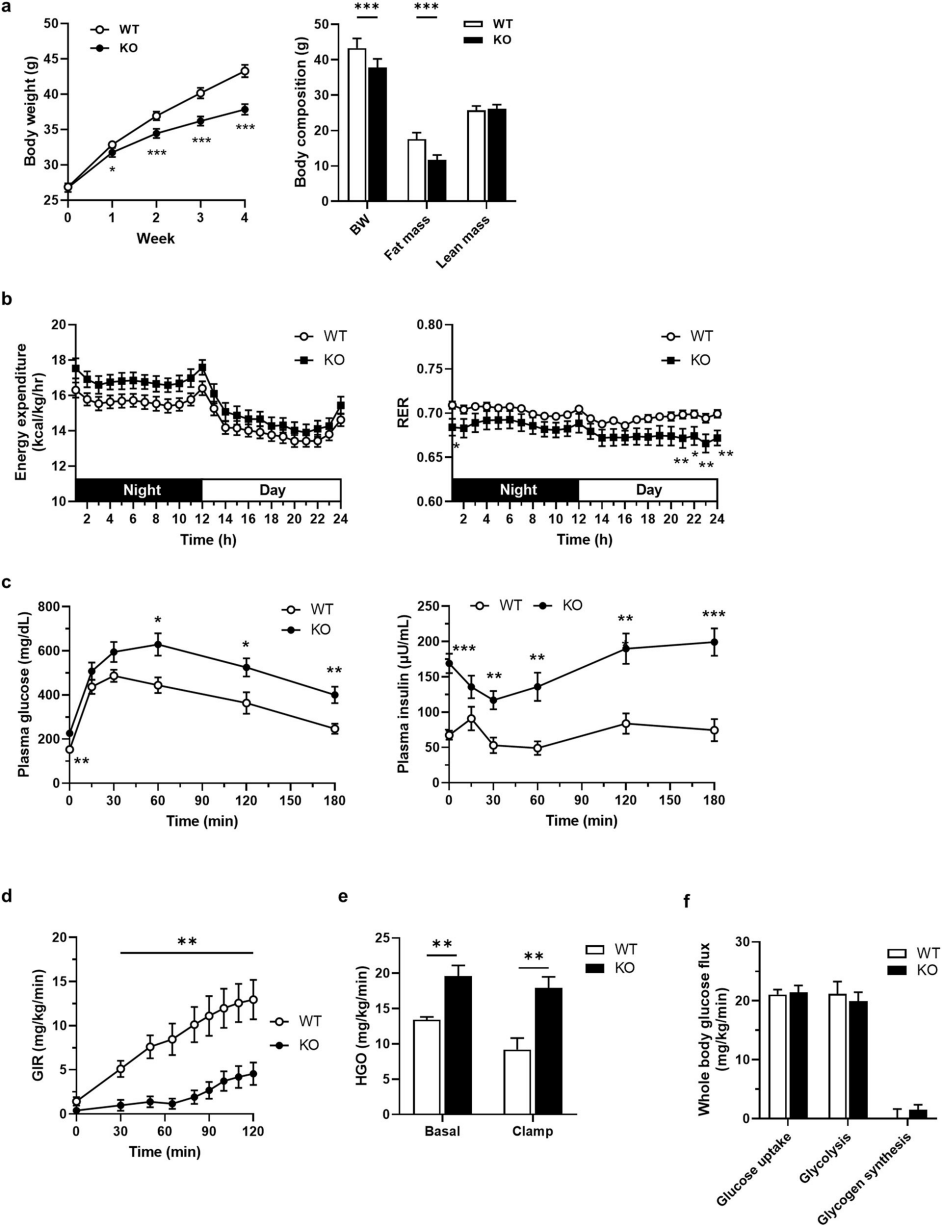
The *Inhbe*-KO mouse has a lean but lipodystrophic phenotype. **a** Changes in the body weights of the *Inhbe* WT and KO mice during HFD feeding for 4 weeks (WT, *n* = 10; KO, *n* = 10). Body composition of the WT and KO mice after HFD feeding for 4 weeks (WT, *n* = 10; KO, *n* = 10). **b** Energy expenditure and RER values of WT and KO mice after HFD feeding for 2 weeks (WT, *n* = 13; KO, *n* = 7). **c** Plasma glucose and insulin levels of WT and KO mice fed a HFD during the glucose tolerance test (WT, *n* = 6; KO, *n* = 8). **d**–**f** Results of hyperinsulinemic–euglycemic clamp after HFD feeding for 4 weeks (WT, *n* = 5; KO, *n* = 6): glucose infusion rate (GIR) (**d**); HGO (**e**); and whole-body glucose flux (glucose uptake, glycolysis and glycogen synthesis) (**f**). The data are presented as the mean ± s.e.m. The results presented were analyzed using the Student’s *t*-test and compared with those of the WT. *P < 0.05, **P < 0.01, ***P < 0.001.

To investigate the in vivo functions of *Inhbe*, we first conducted metabolic phenotyping of *Inhbe*-KO and wild-type (WT) littermate mice, following a previously reported mouse metabolic phenotyping workflow^18^. Despite similar body weights and lean body masses, the whole-body fat mass of the KO mice was small but significantly lower than that of the WT mice fed a regular diet (Supplementary Fig. 5a). To investigate the role of *Inhbe* in the high-fat challenge, mice were fed a HFD for 4 weeks. *Inhbe*-KO mice were resistant to HFD-induced obesity, as reflected by their significantly lower body weight and fat mass (Fig. 2a). To further elucidate the mechanism underlying the lean phenotype of the KO mice, energy balance (energy intake and energy expenditure) was assessed using a metabolic monitoring system after 2 weeks of HFD. While energy intake was similar between the WT and KO mice (Supplementary Fig. 5b), the energy expenditure of the KO mice tended to be higher than that of the WT mice (Fig. 2b). The respiratory exchange ratio (RER), which reflects the relative contribution of carbohydrate or fat oxidation to total energy expenditure, was lower in the KO mice than in the WT mice, indicating a fat preference as an energy source during HFD feeding. These data clearly indicate that *Inhbe*-KO mice exhibit a lean phenotype resulting from increased energy expenditure through preferred fat utilization.

To gain insight into the effects of the lean phenotype of *Inhbe*-KO mice on glucose tolerance, a glucose tolerance test was conducted. Compared with WT mice, HFD-fed KO mice presented higher fasting glucose and insulin levels, indicating impaired glucose tolerance (Fig. 2c and Supplementary Fig. 5c–f). During the test, the higher insulin levels in *Inhbe*-KO mice suggested that the glucose intolerance of *Inhbe*-KO mice resulted from decreased insulin sensitivity. Therefore, we performed a hyperinsulinemic–euglycemic clamp study using an isotope dilution method to obtain further insights into whole-body and tissue-specific glucose metabolism in HFD-fed KO mice. During the clamp test, the glucose infusion rates in the KO mice were dramatically lower than those in the WT mice, indicating severe whole-body insulin resistance in the KO mice (Fig. 2d and Supplementary Fig. 5g,h). The hepatic glucose output (HGO) of the KO mice under both basal and clamp conditions was significantly higher than that of the WT mice (Fig. 2e). Insulin-stimulated suppression of HGO was also decreased in the KO mice (Supplementary Fig. 5i). However, the whole-body glucose uptake, glycolysis and glycogen synthesis rates were similar between the WT and KO groups (Fig. 2f). In addition, through western blotting analysis of insulin-induced Akt phosphorylation in tissues that strongly influence whole-body insulin sensitivity, particularly liver and skeletal muscle, we found that severe insulin resistance occurred only in liver tissue (Supplementary Fig. 5j). These data indicate that *Inhbe*-KO mice exhibit impaired glucose tolerance due to severe hepatic insulin resistance. Finally, TG and total cholesterol levels were significantly increased in the KO mice (Supplementary Fig. 5k). Taken together, our metabolic phenotyping data clearly indicate that *Inhbe*-KO mice have a lean phenotype due to increased energy expenditure through fat oxidation but exhibit a lipodystrophic phenotype under HFD conditions.

### *Inhbe*-KO mice have severe fatty liver

**Fig. 3:**
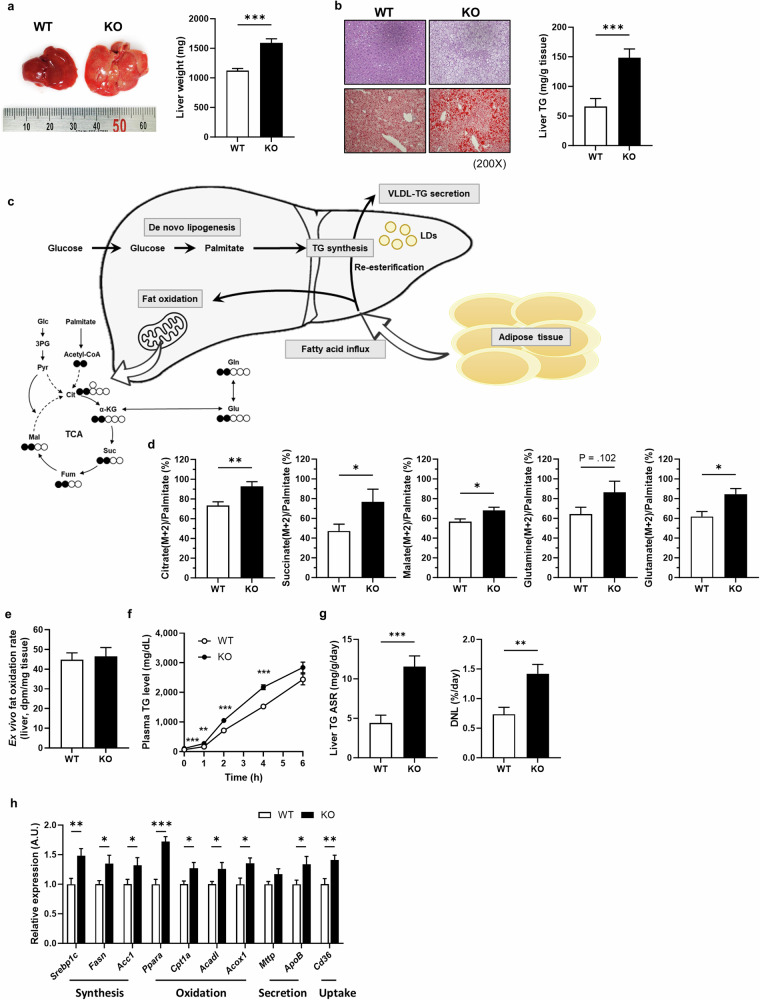
The *Inhbe*-KO mouse has a severe fatty liver phenotype. **a** Representative liver image and weight after HFD feeding for 4 weeks (WT, *n* = 9; KO, *n* = 10). **b** Hematoxylin and eosin (H&E) (top) and Oil Red O (bottom) staining of liver tissue. TG contents in the liver tissue (WT, *n* = 10; KO, *n* = 10). **c** A schematic diagram of hepatic steatosis: fat oxidation, VLDL-TG secretion, TG synthesis and fatty acid influx. **d** The percentage of the ^13^C-labeled TCA intermediate (citrate, succinate, malate, glutamine and glutamate) fraction (M + 2) normalized by enrichment of plasma palmitate in the liver tissues of the WT and KO mice fed a HFD for 4 weeks (WT, *n* = 8; KO, *n* = 5). **e** Ex vivo fat oxidation rate in the liver tissue (WT, *n* = 9; KO, *n* = 9). **f** VLDL secretion rate. Plasma TAG levels after injection of Poloxamer-407 in WT and KO mice fasted overnight (WT, *n* = 10; KO, *n* = 10). **g** Absolute synthesis rates (ASRs) of TG and DNL rates in liver tissue (WT, *n* = 10; KO, *n* = 10). **h** mRNA levels of genes related to lipid synthesis, oxidation, secretion and uptake in the livers of *Inhbe* WT and KO mice fed a HFD for 4 weeks (WT, *n* = 9; KO, *n* = 10). The data are presented as the mean ± s.e.m. The results presented were analyzed using the Student’s *t*-test and compared with those of the WT. *P < 0.05, **P < 0.01, ***P < 0.001.

To understand the lipodystrophic phenotype associated with severe hepatic insulin resistance in *Inhbe*-KO mice, we focused on the livers of these mice. The size and weight of the livers of the KO mice were significantly greater than those of the WT mice (Fig. 3a). Histological analysis also revealed severe fatty liver in the KO mice. The hepatic TG content and plasma ALT levels were also considerably increased in the KO mice (Fig. 3b and Supplementary Fig. 6a).

To reveal the mechanism of hepatic fat accumulation in *Inhbe*-KO mice, we measured metabolic fluxes in vivo and ex vivo after a 4-week HFD (Fig. 3c). First, we measured the in vivo hepatic fat oxidation rate by analysis of ^13^C-labeled tricarboxylic acid (TCA) intermediates after systemic infusion of ^13^C-labeled palmitate (Supplementary Fig. 6b). The ^13^C-labeled TCA intermediates were significantly increased in the KO mice (Fig. 3d), indicating an increased fat oxidation rate in the TCA cycle. However, the ex vivo hepatic fat oxidation rate in liver tissue isolated from HFD-fed KO mice was comparable to that in WT mice (Fig. 3e). These results indicate that the increased hepatic fat oxidation rate resulted from an increased supply of fatty acids rather than a difference in fat oxidation capacity between the WT and KO mice. Next, we analyzed very-low-density lipoprotein (VLDL) secretion following the injection of poloxamer-407 in overnight-fasted mice. VLDL secretion from the liver was greater in the KO mice than in the WT mice (Fig. 3f). However, the increased fat oxidation and VLDL secretion rates made it difficult to explain the severe fatty liver observed in the KO mice. Finally, we measured the fluxes of hepatic TG synthesis and de novo lipogenesis (DNL) using D_2_O labeling for 5 days in vivo (Supplementary Fig. 6c). Hepatic TG synthesis rates dramatically increased in KO mice, which was believed to be responsible for hepatic steatosis in *Inhbe*-KO mice (Fig. 3g). Since the DNL rate accounts for only 1–1.5% of hepatic TG synthesis, the increased hepatic TG synthesis appears to be attributed primarily to elevated re-esterification caused by fatty acid influx from other tissues, especially adipose tissue. Consistently, the expression of genes related to fat synthesis, oxidation, VLDL secretion and uptake was significantly increased (Fig. 3h). These data suggest that severe hepatic steatosis in *Inhbe*-KO mice results primarily from increased fatty acid influx from other tissues.

### Increased lipolysis in adipose tissue causes hepatic fat accumulation in *Inhbe*-KO mice

**Fig. 4:**
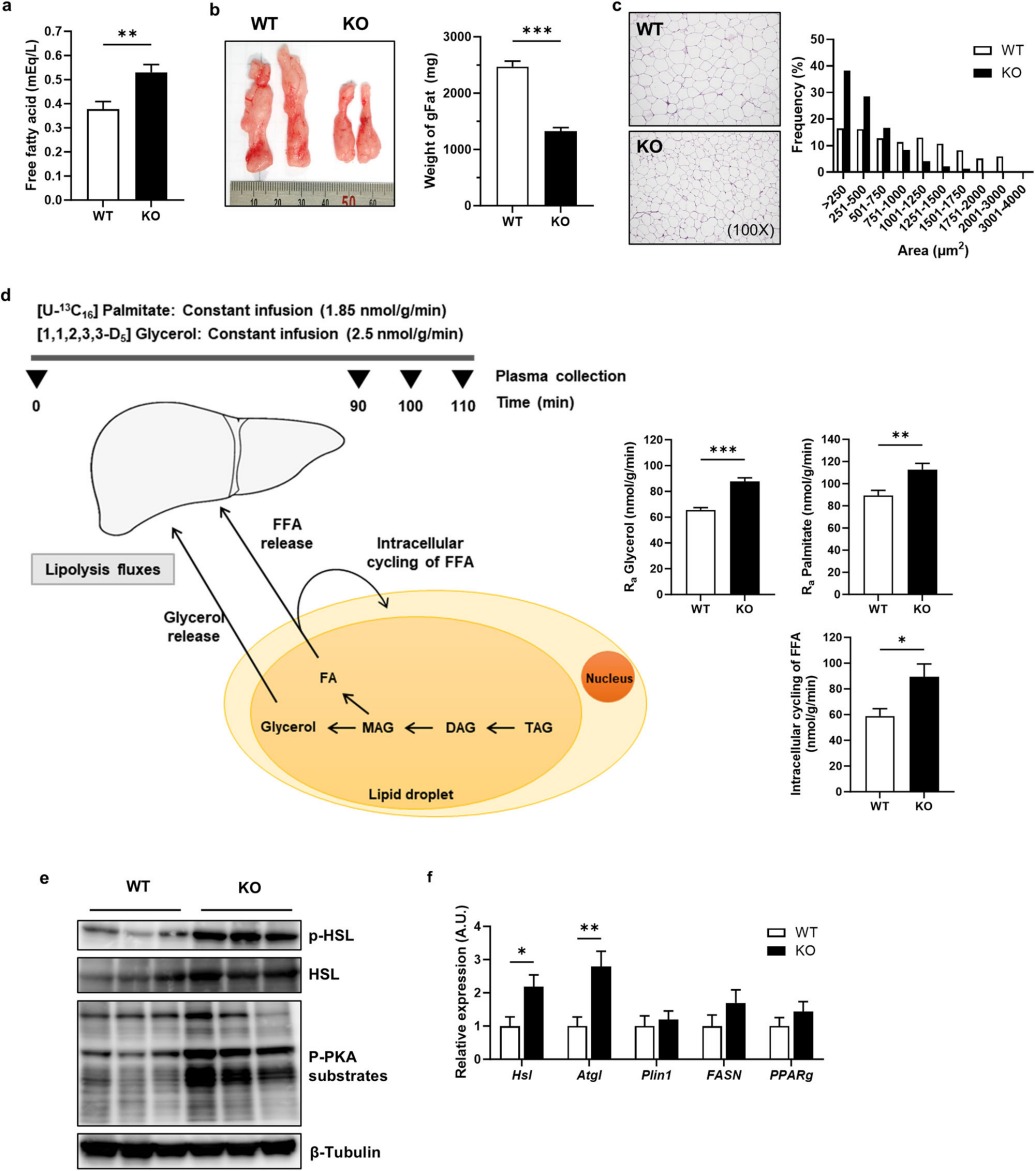
Increased lipolysis in adipose tissue causes hepatic fat accumulation in *Inhbe*-KO mice. **a** Free fatty acid levels in the plasma of WT and KO mice fed a HFD for 4 weeks (WT, *n* = 9; KO, *n* = 10). **b** Tissue image and weight of gFat tissue from WT and KO mice (WT, *n* = 9; KO, *n* = 10). **c** H&E staining of gFat tissue and the distribution profile of adipocyte cell size in gFat tissue. **d** Rates of lipolysis (glycerol Ra, palmitate Ra and intracellular cycling of FFAs) after infusion of D_5_-glycerol and U-^13^C_16_-palmitate in WT and KO mice fed a HFD for 4 weeks (WT, *n* = 8; KO, *n* = 8). Ra, rate of appearance. **e** Western blot analysis of the phosphorylation or expression of HSL and phosphorylated PKA substrates in the gFat tissue of WT and KO mice fed a HFD for 4 weeks. **f** Relative mRNA expression levels of genes related to lipid metabolism in gFat tissue (WT, *n* = 9; KO, *n* = 10). The data are presented as the mean ± s.e.m. The results presented were analyzed using the Student’s *t*-test and compared with those of the WT. *P < 0.05, **P < 0.01, ***P < 0.001.

To confirm the increased fatty acid influx into the liver in KO mice, we measured the plasma levels of free fatty acids and lipolysis after overnight fasting. The plasma levels of free fatty acids were significantly higher in the HFD-fed KO mice than in the WT mice (Fig. 4a). Moreover, the size and weight of gonadal fat (gFat) were significantly reduced in the KO mice (Fig. 4b), accompanied by a reduction in adipocyte cell size compared with their WT littermates (Fig. 4c). To elucidate the small adipose tissue and increased plasma levels of free fatty acids, lipolysis flux was assessed in vivo by an infusion of D_5_-glycerol and ^13^C-labeled palmitate. The turnover rates of glycerol and palmitate in plasma increased, indicating a marked increase in whole-body lipolysis in *Inhbe*-KO mice (Fig. 4d). Consistent with the increased lipolysis in vivo, the signaling pathways and gene expression levels related to lipolysis were markedly activated in the adipose tissue of the KO mice (Fig. 4e,f). These findings demonstrate that activin E deficiency increases lipolysis and suggest that activin E plays a suppressive role in lipolysis in adipose tissue.

### Activin E suppresses lipolysis in adipocytes via the ALK7–Smad pathway

**Fig. 5:**
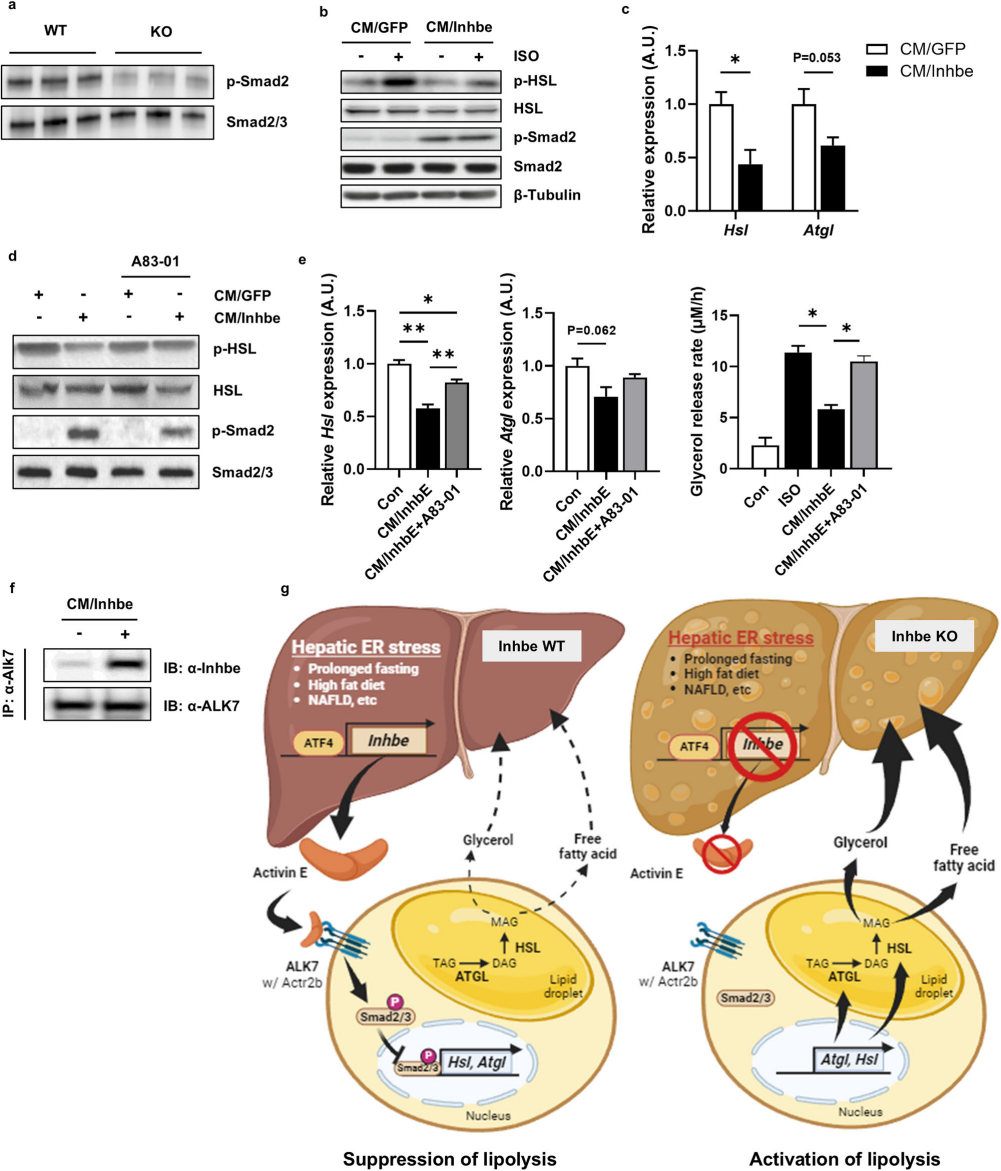
Activin E suppresses lipolysis in adipocytes via the ALK7–Smad pathway. **a** Western blot analysis of the phosphorylation of Smad2 in *Inhbe* WT and KO mice fed a HFD for 4 weeks. **b** Western blot analysis for the phosphorylation of HSL in fully differentiated C3H10T1/2 adipocytes treated with conditioned media from cells overexpressing *Inhbe* with or without 1 μM isoproterenol (ISO). **c** Relative mRNA expression levels of *Hsl* and *Atgl* in fully differentiated C3H10T1/2 adipocytes treated with conditioned media from cells overexpressing *Inhbe*. **d** Western blot analysis of the phosphorylation of HSL and Smad2 in fully differentiated C3H10T1/2 adipocytes treated with conditioned media (CM/GFG and CM/Inhbe) from hepatocytes overexpressing *Inhbe* with or without 1 μM A83-01. **e** Relative mRNA expression levels of *Hsl* and *Atgl* and ISO-induced glycerol release in fully differentiated C3H10T1/2 adipocytes treated with conditioned media from cells overexpressing *Inhbe* with or without 1 μM A83-01. **f** Western blot analysis of *Inhbe* in the cell lysates immunoprecipitated with anti-ALK7 antibody from differentiated C3H10T1/2 adipocytes treated with conditioned media from cells overexpressing *Inhbe*. IP, immunoprecipitation; IB, immunoblot. **g** A schematic diagram of the mode of action by which activin E regulates adipose tissue lipolysis (*Inhbe* WT, left; *Inhbe* KO, right).

Proteins belonging to the TGF-β superfamily generally act through the Smad pathway. To understand the regulatory mechanism of activin E in adipose tissue lipolysis, we investigated the Smad pathway in adipose tissue. Smad2 phosphorylation markedly decreased in the adipose tissue of the KO mice (Fig. 5a). We also confirmed that Smad2 phosphorylation increased in fully differentiated C3H10T1/2 cells treated with conditioned media from *Inhbe*-overexpressing cells (CM/Inhbe) (Fig. 5b). In addition, *Hsl* and *Atgl* mRNA levels were reduced following CM/Inhbe treatment (Fig. 5c).

A recent report suggested that ALK7, which is predominantly expressed in white adipose tissue, can regulate lipolysis^19–21^. Moreover, activin B secreted from adipocytes under fed conditions has been reported to directly bind to ALK7, activating the Smad2 pathway to suppress the expression of lipolytic genes. Therefore, we tested whether *Inhbe* regulated lipolysis via the ALK7–Smad pathway. A83-01, a selective inhibitor of ALK7, suppressed CM/Inhbe-induced Smad2 phosphorylation (Fig. 5d). The decreased expression of lipolytic genes and glycerol release induced by CM/Inhbe were reversed by A83-01 treatment (Fig. 5e). Finally, we confirmed direct binding between activin E and ALK7 using coimmunoprecipitation (Fig. 5f). Collectively, these results indicate that activin E acts as a negative regulator of lipolysis via the ALK7–Smad2 pathway in white adipose tissue.

### Overexpression of activin E improves the fatty liver of *Inhbe*-KO mice by suppressing lipolysis in adipose tissue

**Fig. 6:**
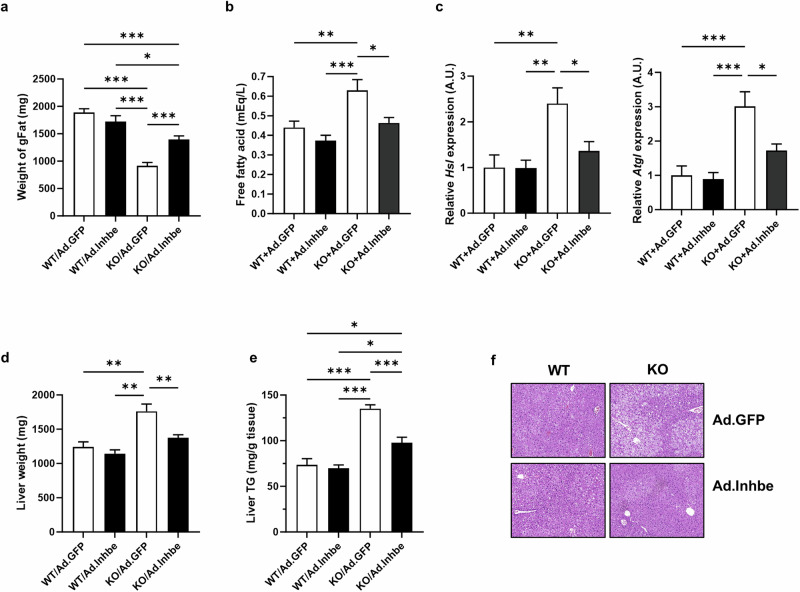
Overexpression of activin E improves the fatty liver of *Inhbe*-KO mice by suppressing lipolysis in adipose tissue. **a** Tissue weights of gFat tissue from WT and KO mice fed a HFD for 3 weeks with adenoviral overexpression of *Inhbe* (WT+Ad/GFP, *n* = 6; WT+Ad/Inhbe, *n* = 5; KO+Ad/GFP, *n* = 8; KO+Ad/Inhbe, *n* = 9). **b** Plasma free fatty acid levels in WT and KO mice fed a HFD for 3 weeks with adenoviral overexpression of *Inhbe* (WT+Ad/GFP, *n* = 5; WT+Ad/Inhbe, *n* = 6; KO+Ad/GFP, *n* = 5; KO+Ad/Inhbe, *n* = 6). **c** Relative mRNA expression levels of *Hsl* and *Atgl* in gFat tissue from WT and KO mice fed a HFD for 3 weeks with adenoviral overexpression of *Inhbe* (WT+Ad/GFP, *n* = 9; WT+Ad/Inhbe, *n* = 9; KO+Ad/GFP, *n* = 10; KO+Ad/Inhbe, *n* = 9). **d** Liver tissue weights of WT and KO mice fed a HFD for 3 weeks with adenoviral overexpression of *Inhbe* (WT+Ad/GFP, *n* = 6; WT+Ad/Inhbe, *n* = 5; KO+Ad/GFP, *n* = 9; KO+Ad/Inhbe, *n* = 8). **e** Representative H&E staining images of liver tissue from WT and KO mice fed a HFD for 3 weeks with adenoviral overexpression of *Inhbe*. **f** TG contents in liver tissue from WT and KO mice fed a HFD for 3 weeks with adenoviral overexpression of *Inhbe* (WT+Ad/GFP, *n* = 6; WT+Ad/Inhbe, *n* = 5; KO+Ad/GFP, *n* = 7; KO+Ad/Inhbe, *n* = 9).

To confirm whether activin E could reverse hepatic steatosis in *Inhbe*-KO mice by suppressing lipolysis in adipose tissue, we overexpressed *Inhbe* in both WT and KO mice via the adenoviral system (Ad.GFP and Ad.Inhbe). Although there was little effect on the weight of gFat in the WT mice due to the relatively short treatment period, the overexpression of *Inhbe* significantly reversed the weight of gFat in the KO mice (Fig. 6a). Free fatty acid levels were also reduced in Ad.Inhbe-administered KO mice to levels similar to those in WT mice (Fig. 6b). Moreover, overexpression of *Inhbe* significantly reduced the expression levels of the adipose lipolytic *Hsl* and *Atgl* genes (Fig. 6c). The weight and TG content of the liver in Ad.Inhbe-administered KO mice were also significantly reduced to levels similar to those in WT mice (Fig. 6d,e). Histological analysis confirmed an improvement in fatty liver following *Inhbe* overexpression in KO mice (Fig. 6f). These data suggest that activin E functions as a protective hepatokine, preventing excess fat accumulation by suppressing adipose lipolysis.

## Discussion

In the present study, we investigated the precise mechanisms underlying the regulation of activin E expression in the liver and its function in NAFLD progression. Activin E is a hepatokine primarily expressed and secreted by the liver, and its expression is upregulated by ATF4 under conditions such as hepatic ER stress, prolonged fasting, HFD and NAFLD. Secreted activin E circulates systemically and mainly suppresses adipose tissue lipolysis via ALK7, leading to reduced fat mobilization and hepatic fatty acid influx from adipose tissue. Activin E binds directly to ALK7, which is expressed predominantly in adipose tissue, activating the Smad2 signaling pathway and suppressing the expression of lipolytic genes, such as *Hsl* and *Atgl*. Therefore, the deficiency of *Inhbe* resulted in increased lipolysis in adipose tissue, leading to increased fatty acid influx into the liver. Conversely, the overexpression of activin E improves fatty liver by suppressing lipolysis in adipose tissue. Collectively, activin E may function as a protective hepatokine, preventing excess fat accumulation by suppressing adipose lipolysis through a feedback control mechanism. A schematic diagram summarizing the role of activin E in regulating adipose tissue lipolysis is shown in Fig. 5g.

During fasting, elevated levels of plasma fatty acids derived from adipose tissue are used as an energy source in the liver^22,23^. However, prolonged fasting can lead to excessive lipid influx into the liver, disturbing protein and lipid metabolism and triggering hepatic ER stress^15^. Several studies have shown that hepatic ER stress induced by prolonged fasting activates the ER stress response to overcome stress. For example, FGF21, which is induced by the ER stress response during prolonged fasting, can reduce lipid accumulation by promoting fat oxidation via the peroxisome proliferator-activated receptor (PPAR)-α pathway in the liver^6^. In this study, we observed increased *Fgf21*, *Atf4* and *Inhbe* mRNA levels in the liver during fasting^16^. While the expression of *Fgf21* increased with the accumulation of hepatic TGs, the induction of *Inhbe* expression was delayed until the later stages of fasting. On the basis of these findings, we suggest that compensatory mechanisms for hepatic fat accumulation involve primarily the activation of fat oxidation through FGF21 induction and secondarily the reduction in fatty acid influx to the liver by activin E secretion.

We also discovered a mechanism underlying the induction of *Inhbe* expression in hepatic steatosis. Previous studies have reported that *Inhbe* expression is upregulated by ATF4 under chemically induced ER stress^13^. In this study, ATF4 was identified as a key factor in the expression of *Inhbe* in response to hepatic steatosis both in vivo and in vitro. We demonstrated that direct binding of ATF4 to the proximal region of the promoter was critical for *Inhbe* expression under hepatic steatosis conditions. These data indicate that ATF4 acts as a primary transcriptional regulator of *Inhbe* expression under ER stress.

Previous reports have suggested that insulin induces hepatic *Inhbe* expression under hyperinsulinemic conditions, such as HFD conditions, *db/db* mouse models, and insulin resistance^8,12^. These reports suggest that insulin directly induces *Inhbe* expression via C/EBPs in cultured hepatocytes. However, hepatic *Inhbe* expression was higher in fasting conditions than in feeding conditions in an in vivo fast-refeeding study^24^. Even in a report suggesting insulin-dependent *Inhbe* expression in hepatocytes, hepatic *Inhbe* expression during fasting was greater than that in the fed state^8^. We also observed elevated hepatic *Inhbe* expression during fasting (Fig. 1). Moreover, hepatic *Inhbe* expression was more strongly correlated with *Atf4* expression than with *Cebpb* expression in our GEO database analysis of NAFLD (Fig. 1 and Supplementary Fig. 2). Fat accumulation in the liver can induce hepatic ER stress, resulting in hepatic insulin resistance, which is typically accompanied by hyperinsulinemia. Therefore, elevated *INHBE* expression in humans with insulin resistance may be caused by hepatic ER stress rather than hyperinsulinemia. Moreover, we confirmed that increased *Inhbe* expression under HFD feeding was suppressed by ER stress inhibitors alone under in vivo and in vitro conditions. Therefore, hepatic ER stress is a more critical inducer of *Inhbe* expression than hyperinsulinemia is. Another study suggested that hepatic *Inhbe* expression may increase due to PPAR-α activation by increased fatty acid influx into the liver^25^. However, we could not identify any putative PPAR-response elements in the proximal promoter of *Inhbe* within approximately −1,200 to +1 nt (data not shown). Furthermore, we observed a negative correlation between *Inhbe* and *Ppara* expression in the GEO database analysis of NAFLD (Supplementary Fig. 2c,d). One possible hypothesis for hepatic *Inhbe* induction by PPAR-α is that it may be mediated through CREB3L3, known as CREBH. Under fasting and ER stress conditions, PPAR-α and CREB3L3 are known to be key players in regulating liver metabolism^26^. PPAR-α activated by fatty acid influx induces CREB3L3 transcription, and CREB3L3 may act as a transcription factor for hepatic *Inhbe* expression. CREB3L3 binds to and induces a cAMP response element in the promoters of target genes. ATFs and CREBs belong to the ATF/CREB family and share the consensus DNA-binding element 5′-TGACGXAAX-3′. The putative ATF4 binding element in the proximal promoter of *Inhbe* may be a cAMP response element for CREB3L3 binding. In addition, CREB3L3 overexpression in AML12 cells induced *Inhbe* expression (Supplementary Fig. 2g). However, further studies are needed to elucidate the molecular regulatory mechanisms underlying CREB3L3-induced *Inhbe* expression in vivo and in vitro.

Several studies have suggested that activin E plays a metabolic role in addition to its predominant expression in the liver. Liver-specific overexpression of *Inhbe* enhances energy expenditure by browning inguinal white adipose tissue^27^. Treatment with *Inhbe* small interfering RNA (siRNA) in obese and diabetic *db*/*db* mice substantially reduces adiposity and increases lipid utilization^12^. Recent studies have suggested that activin E can suppress adipose lipolysis by measuring free fatty acid and glycerol release from adipocytes ex vivo and in vitro^24^^,28^. In this study, we demonstrated that activin E plays a major role in the suppression of adipose lipolysis through direct assessment of in vivo glycerol and lipid fluxes with stable isotopes and analysis of lipolytic gene expression in adipose tissue. So far, no direct mediator regulating activin E-mediated lipolysis has been identified. Here, we propose that activin E regulates lipolysis through the ALK7–Smad2 signaling pathway. ALK7, also known as activin A receptor type 1c (ACVR1c), is a receptor tyrosine kinase and belongs to the receptor for the TGF-β superfamily. ALK7 has multiple physiological functions, including metabolic regulation, macrophage activation and tumor suppression, primarily through the Smad2/3 signaling pathway^29–31^. In human GWASs, rare *INHBE* loss-of-function variants and variants in *ACVR1C* (ALK7) have been associated with a lower waist-to-hip ratio and regulation of fat distribution^32,33^. *Alk7*-KO mice have reduced body weight and fat mass, which is attributed to increased lipolysis in adipose tissue^21,34^. In addition, other activin family proteins, such as activins B and C, can directly bind to ALK7^35,36^. Recent studies have suggested that the suppressive effect of activin E on lipolysis in adipose tissue may be mediated by ALK7 through the use of *Acvr1c*-null mice, ACVR1C monoclonal antibody and *Acvr1c* knockdown^24,28^. However, there is no direct evidence of binding between activin E and ALK7. In our study, we demonstrated that activin E directly binds to ALK7 and suppresses lipolysis via the ALK7–Smad2 pathway, as shown by analyses of lipolytic gene expression and glycerol release in adipocytes after treatment with CM/Inhbe and an ALK7 inhibitor (Fig. 5). Furthermore, considering the expression pattern of ALK7, which is expressed primarily in adipose tissue, and the metabolic phenotypes of *Inhbe*-KO mice, adipose tissue seems to be the primary target of activin E, whereas the alterations observed in other tissues, particularly the liver, are likely to be secondary outcomes.

We also observed that *Inhbe*-KO mice presented reduced body weight and fat mass, which can be attributed to increased whole-body energy expenditure. The increased energy expenditure in *Inhbe*-KO mice appears to be due to increased fat burning in the liver (Fig. 3) and/or adipose tissue. Increased hepatic fat oxidation occurs secondarily to the increased fat influx into the liver, driven by increased adipose tissue lipolysis, as demonstrated by metabolic flux studies using stable isotopes. In addition, the elevated monoacylglycerol levels within adipocytes resulting from increased adipose tissue lipolysis may activate PPAR-α, thereby promoting mitochondrial energy expenditure in adipose tissue^37^. Furthermore, several studies have shown that increased adipose tissue lipolysis can lead to greater fat utilization and energy expenditure^38–42^.

In human GWASs, predicted partial loss-of-function variants in *INHBE* are associated with reduced visceral white adipose tissue and improved metabolic parameters, including a lower incidence of diabetes and serum TG levels^32,33^. However, human metabolic phenotypes differ from those observed in recent rodent studies. Studies involving the genetic deletion of *Inhbe*, including ours, have consistently shown similar metabolic phenotypes: lean, elevated plasma-free fatty acids, glucose intolerance and insulin resistance (Figs. 2 and 3)^24,28^. These differences between the metabolically sensitive phenotypes in humans and the lipodystrophic phenotypes in mice appear to be attributable to differences in fat distribution and hepatic lipid metabolism^43^. Mouse models often show rapid liver lipid accumulation under HFDs, leading to pronounced lipodystrophy, whereas in humans, hepatic lipid accumulation occurs more gradually and is influenced by factors such as insulin resistance and subcutaneous fat storage capacity. This contributes to distinct metabolic responses, where mice tend to exhibit more extreme lipid dysregulation in the liver than do humans under similar conditions. For example, mice have high high-density lipoprotein (HDL) and low VLDL and low-density lipoprotein (LDL) levels due to a lack of cholesteryl ester transfer protein, whereas humans have high VLDL and low-density lipoprotein levels^44^. Therefore, if the hepatic capacity for VLDL secretion (transporting of liver fat to peripheral tissues) or mitochondrial fat oxidation is greater in humans than in mice, these differences in hepatic lipid metabolism may contribute to species-specific differences in metabolic phenotypes. This is especially relevant given that excess free fatty acids from increased lipolysis, due to the lack of *Inhbe*, enter the liver.

Hepatic steatosis can arise from decreased hepatic fat oxidation and VLDL secretion, increased TG synthesis and DNL levels, and increased free fatty acid influx^45^. In this study, we dissected each metabolic flux contributing to hepatic steatosis using stable isotopes. (1) Increased fat oxidation rate: although the in vivo hepatic fat oxidation rate increased, ex vivo fat oxidation in the liver tissue remained comparable between WT and KO mice. These results imply that the increased fat oxidation rate in vivo resulted from an increased supply of substrates rather than an increased fat oxidation capacity of the liver itself. Considering the increased fat oxidation, this was unlikely to be the cause of hepatic fat accumulation. (2) Increased VLDL secretion rate: increased VLDL secretion contrasts with hepatic steatosis. (3) Increased TG synthesis and DNL rates: increased hepatic TG synthesis and DNL rates may cause hepatic steatosis. Augmented hepatic TG synthesis may be accelerated by an increased influx of fatty acids into the liver from other sources rather than a direct effect of activin E, given the very low expression of ALK7 in hepatocytes. Moreover, increased DNL is less likely to contribute meaningfully to hepatic fat accumulation, as it was shown to contribute only 1–1.5% to hepatic TG synthesis. Therefore, increased fat oxidation and VLDL secretion rates appear to be compensatory mechanisms against hepatic TG accumulation induced by excess fatty acid influx. Collectively, given that the loss of activin E results in severe fatty liver from excess adipose lipolysis, all these direct pieces of evidence, including gene regulation in the liver, receptor binding and action in adipose tissue, and hepatic metabolic fluxes, strongly indicate that activin E may function as a protective hepatokine, preventing NAFLD through the suppression of lipolysis.

Various therapeutic strategies have been proposed for the treatment of obesity and NAFLD. Among these approaches, the regulation of adipose lipolysis has recently emerged as a target for obesity and NAFLD treatment. First, inhibiting adipose lipolysis to reduce circulating free fatty acids and their influx into the liver may be an effective strategy for treating hepatic steatosis and insulin resistance. We confirmed the hepatoprotective effects of adenovirus-overexpressing *Inhbe*, which is consistent with previous studies^28^. These results highlight that activin E is an attractive therapeutic target for protection against hepatic steatosis. However, it is important to consider the potential risks of unhealthy adipocyte expansion due to the inhibition of lipolysis in adipocytes, resulting in adipose inflammation and obesity. Therefore, long-term investigations into lipolysis inhibition for hepatic steatosis treatment should be considered. Second, it can be speculated that activating lipolysis in adipose tissue could be beneficial for controlling obesity, rather than NAFLD, through *Inhbe* inhibition. However, it is crucial to consider that increased levels of circulating free fatty acids resulting from the activation of lipolysis can induce ectopic fat accumulation, ER stress and insulin resistance in various peripheral tissues, especially the liver. This phenomenon is frequently observed in mouse models, as observed in our study and in previous reports^45,46^. Nevertheless, given the correlation between the partial loss of function of *INHBE* and reduced visceral adiposity risk of metabolic disease in human GWASs, a potential strategy could involve simultaneously activating both adipose lipolysis and fat burning in peripheral tissues.

In summary, this study provides insights into the mechanism responsible for *Inhbe* expression under NAFLD conditions, involving the activation of ATF4 induced by ER stress. Our results clearly demonstrate that activin E inhibits lipolysis by activating ALK7–Smad signaling in adipose tissue, leading to reduced fat mobilization and hepatic fatty acid influx. These findings strongly suggest that activin E serves as a regulator that prevents the influx of fatty acids into the liver, ultimately offering protection against NAFLD.

## Supplementary information

Supplementary Information
