# Supplementary material for: Activin E is a new guardian protecting against hepatic steatosis via inhibiting lipolysis in white adipose tissue
Source: Exp Mol Med. 2025 Feb 13;57(2):466–77. doi: 10.1038/s12276-025-01403-6 (PMC11873131; doi:10.1038/s12276-025-01403-6)
Supplement: Supplementary file 1 — Supplementary Information [file 12276_2025_1403_MOESM1_ESM.pdf]

Supplementary Information

Activin E is a new guardian protecting against hepatic steatosis via inhibiting lipolysis in white adipose tissue

Shi-Young Park[1,2,11], Yoonil Cho[1,3,11], Sae-Mi Son[1,3], Jang Ho Hur[1], Yeongmin Kim[3,4], Hyunhee Oh[1,2], Hui-Young Lee[1,8], Sungwon Jung[5,6], Sanghee Park[4,7], Il-Young Kim[1,4,8], Se-Jin Lee[9], and Cheol Soo Choi[1,8,10]

Correspondence Cheol Soo Choi, Endocrinology, Internal Medicine, Gachon University Gil Medical Center, Incheon 21565, Korea. Email: cschoi@gachon.ac.kr.

The file includes

1. Supplementary Materials and Methods
2. Supplementary Figures 1 - 6

Supplementary Materials and Methods

Clinical relevance analysis of *INHBE*

We analyzed five transcriptome datasets from the Gene Expression Omnibus (GEO) public database of the National Center for Biotechnology Information (NCBI) (GSE105127, GSE130970, GSE135251, GSE162694) and from the European Nucleotide Archive (ENA) public database (PRJNA512027) of the European Bioinformatics Institute (EMBL-EBI). After normalization among five datasets, TPM values of *INHBE* and potential transcription factors including *ATF4* were compared according to the NAFLD status (Normal, steatosis, and NASH). Correlation of *INHBE* expression with potential transcription factor expressions were analyzed by Pearson correlation with GraphPad Prism ver.10.

Generation of *Inhbe* KO mice

For generation of mice carrying a deletion of the *Inhbe* coding sequence, a targeting construct was prepared containing: (i) an approximately 6 kb segment of the *Inhbe* locus up the *Hin*dIII site immediately after the start codon, (ii) a cassette containing the coding sequence for *Lac*Z and processing signals derived from SV40, (iii) a PGKneo expression cassette, and (iv) an approximately 2 kb segment of the *Inhbe* locus extending 3' from the first *Eco*RI site downstream of the coding sequence. Correctly targeted embryonic stem cells were then used to generate mice carrying the modified allele lacking the entire Inhbe coding sequence. Mice in which the targeted allele was transmitted through the germline were then backcrossed at least 6 times onto a C57BL/6 genetic background.

Adenoviral study

Adenovirus overexpressing *Inhbe* and GFP were purchased VECTOR BIOLABS (PA, USA). Viruses were diluted in PBS and administered at dose of 1 x 10^9 pfu/mouse via tail-vein.

Body composition and analysis of energy balance

Whole-body fat and lean masses were measured through ^1H magnetic resonance spectroscopy (LF-90II, Bruker Optics, Germmany). After a one-day acclimation, O_2

consumption (VO_2), CO_2 production (VCO_2), locomotor activity, and food/water consumption were measured for 48 hours in a metabolic monitoring system (comprehensive animal metabolic monitoring system, CLAMS, Columbus Instruments, Columbus, USA). Energy expenditure and respiratory exchange ratio (RER) were calculated from the gas exchange data: RER as the ratio of VCO_2 to VO_2, Energy expenditure = (3.815 + 1.232 × RER) × VO_2.

Glucose tolerance test (GTT)

For the IPGTTs, mice were fasted for 16 hr, and glucose (1.5 g/kg body weight) was injected intraperitoneally. Blood glucose and insulin levels were measured at 0, 15, 30, 60, 120, and 180 min after glucose injection with a glucose analyzer (GM9, Analox Instruments Ltd., UK) and Rat Insulin RIA kit (Merck Millipore, Darmstadt, Germany).

Hyperinsulinemic-euglycemic clamp study with isotope dilution method

Calculation of glucose flux: For the determination of plasma ^{3}H-glucose, plasma was deproteinized with $ZnSO_4$ and $Ba(OH)_2$, dried to remove ^3H_2O, resuspended in water, and counted in scintillation fluid (Ultima Gold; PerkinElmer) on a scintillation counter (Beckman, Fullerton, CA). Rates of basal and insulin-stimulated whole-body glucose turnover were determined as the ratio of the [3-^{3}H]glucose infusion rate (dpm) to the specific activity of plasma glucose (dpm per milligram) at the end of the basal period and during the final 30 min of the clamp experiment, respectively. Hepatic glucose production was determined by subtracting the glucose infusion rate from the total glucose appearance rate. The plasma concentration of ^3H_2O was determined by the difference between ^{3}H counts without and with drying. Whole-body glycolysis was calculated from the rate of increase in plasma ^3H_2O concentration divided by the specific activity of plasma ^{3}H-glucose, as described previously (14). Whole-body glycogen synthesis was estimated by subtracting whole-body glycolysis from whole-body glucose uptake, assuming that glycolysis and glycogen synthesis accounted for the majority of insulin-stimulated glucose uptake (14).

Stable isotope metaboic flux study

Stable isotope tracer infusion study: Frozen liver and plasma were dissolved in 70% acetonitrile for determination of tracer enrichment of metabolites. Frozen livers were

ground by using a TissueLyser II (QIAGEN, Germantown, MD, USA). After centrifugation, the supernatant was dried and derivatized with methoxylamine hydrochloride (MOX) and MTBSTFA + 1% TBDMS (Sigma-Aldrich, USA) to measure tracer enrichment of TCA cycle intermediates, glycerol, and palmitate. The derivatized samples were analyzed using gas chromatography mass spectrometry (GC-MS, Agilent, USA) to monitor specific ions associated with the metabolites (1). Mass isotopomer distributions were corrected for natural abundance. Metabolic kinetics were calculated as follows. 1) Rate of appearance of tracee: respective tracer infusion rates (*F*) were divided by isotopic enrichment at plateau to determine rate of appearance (R_a) of tracee (i.e., palmitate, and glycerol). Tracer enrichment of palmitate and glycerol was expressed as mole percent excess (*MPE*), which is calculated as TTR/(1+TTR) where TTR is tracer to tracee ratio (2). 2) Fractional contribution of palmitate flux to TCA cycle: Contribution of palmitate flux to TCA cycle in the designated tissues was quantified as citrate enrichment (M+2) in the tissue normalized by plasma palmitate enrichment. Fully transferred palmitate gives an increase in the enrichment of citrate. 3) Rates of triacylglycerol-fatty acid futile cycling: Futile cycling occurs between triacylglycerol (TG) and FFA in intra-adipocytes (3). Briefly, calculation of intracellular cycling rate (i.e., adipocyte re-esterification after lipolysis) was determined based on R_a palmitate and R_a glycerol, latter of which directly reflects rate of lipolysis. Fractional contribution (FC) of palmitate to total free fatty acid (FFA) is 0.65 at rest.

D_2O labeling study: Liver samples were processed in a chloroform and methanol mixture and H_2O, which were ground, centrifuged and the polar fraction extracted for analysis. After drying under SpeedVac (#SPD111V, Savant Instruments, Farmingdale, NY, USA), samples were prepared for thin layer chromatography (TLC) and visualized under UV light after rhodamine-6G staining. TG spots were extracted, saponified to glycerol and fatty acids (i.e., palmitate), which were derivatized with MOX and MTBSTFA+1%TBDMS. The derivatives were analyzed via GC-MS for metabolites enrichment, corrected for natural abundance. Body water enrichment used the acetone exchange method, analyzing urine or plasma for D_2O enrichment by mass to charge ratios. To determine the enrichment of TG-bound glycerol and palmitate, the ratio of labeled (M+1) to unlabeled (M+0) mass isotopomers were used. Fractional synthesis rate (FSR) of TG and DNL was assessed by using the ratio of precursor (i.e.,

labeling body water) to product (i.e., glycerol or palmitate) and the maximal theoretical number (n) of exchanging H atoms from body water into C-H bonds (i.e., glycerol: 4.7 and palmitate: 22) (4,5). The FSR was calculated as the product enrichment divided by the precursor enrichment. Absolute synthesis rate (ASR) of liver TG was determined by the FSR multiplied by the pool size of TG in liver.

For the stable isotope tracer infusion study and the D_2O labeling study, measurements of stable Isotopic enrichment and calculations of metabolite kinetics were performed by Myocare (Myocare Inc., Korea).

Ex vivo fatty acid oxidation

Liver tissues were freshly removed from mice and were placed in flasks fitted with center wells to trap $^{14}CO_2$. Tissues were incubated in 3 mL Krebs - Ringer phosphate buffer with 2 μC_i of [1-^{14}C] oleic acid (PerkinElmer) and cold oleic acid (0.6 mM final concentration, Sigma) in complex with bovine serum albumin for 30 min at 37°C. Then, 1 mL of 0.5 N sulfuric acid was injected into the media to stop the fatty acid oxidation. Flasks were maintained at 50°C for 3 h to release and evaporation of $^{14}CO_2$ from the media to NaOH solution in the center well. After 3 hr−incubation, contents of the center well were transferred to scintillation fluid and counted radioactivity with liquid scintillation counter (Tri−Carb 3110 TR, PerkinElmer, USA).

Hepatic VLDL secretion assay

Mice were fasted for 4 hours before initiation of VLDL secretion assay. After baseline blood collection (~50ul) from the tail vein, mice were intraperitoneally injected with 10% (w/v) of poloxamer-407 (P-407) (Sigma-Aldrich, USA) at a dose of 1.0 g/kg of body weight. After 1, 2, 4 and 6 hours, approximately 50ul of blood was collected from the tail vein. TG in plasma samples were measured by enzymatic quantification assay kits purchased from Wako Diagnostics (L-type Triglyceride M, GPO-HMMSP, #461-08992/461-090902, Wako, Japan).

Measurement of liver TG contents

Lipids from liver were extracted using the Folch method and measured by enzymatic method using a triglyceride reagent (L-type Triglyceride M, GPO-HMMSP, #461-08992/461-090902, Wako, Japan).

Histology

Liver and gonadal adipose tissues were fixed in 10% neutral-buffered formalin (BBC Biochemical, USA), processed with a standard paraffin-embedding, cut into 5 μm thick sections, and mounted onto slides. The H&E staining and oil-red O staining were carried out by the Core Facility for Cell to In-vivo Imaging at Gachon University. Afterwards, the stained images were digitized using a scanner (PANNORAMIC SCAN II, 3D Histech Ltd., Hungary). For this research, the stained images were randomly selected and digitized for each animal under a 10X objective using the Pannoramic Viewer. The adipocyte sizes and numbers were measured and quantified using the Adiposoft (ver 1.15) plug-in in the ImageJ software.

Cell culture and reagents

C3H10T1/2 cells were purchased from American Type Culture Collection (ATCC, USA) and maintained in Dulbecco's modified Eagle's medium (DMEM; Life Technologies, USA) supplemented with 10% (vol/vol) fetal bovine serum (FBS; Life Technologies, USA) and 1% penicillin/streptomycin. To test adipocyte differentiation, C3H10T1/2 cells seeded and grow until reaching confluence, adipocyte differentiation was induced in these cells with an adipogenic cocktail containing 10 ug/ml of insulin, 1 uM of dexamethasone, 0.5 mM of IBMX. Forty-eight hours after induction, the cells were maintained in DMEM containing 10% FBS, 10 ug/ml of insulin for 12 days. Insulin, dexamethasone, IBMX were obtained from Sigma (St Louis, USA). For conditioned medium preparation, HEK293A cells were seeded in T75 flasks in DMEM supplemented with 10% (vol/vol) fetal bovine serum (FBS; Life Technologies, USA) and 1% penicillin/streptomycin. Then, the cells were transfected with pCMV6/Inhbe plasmid (Origene, USA). After 48 hr incubation after transfection, the culture medium was collected and clarified by centrifugation at 1,000 xg for 3 min. The conditioned medium was concentrated by Amicon Ultra-15 with 30 kDa nominal molecular weight limit (Millipore, USA) and stored at -80°C until use.

RNA isolation and quantitative real-time PCR

Total RNA from cells or tissues was isolated by using a TRIzol reagent extraction kit (Life Technologies, USA) according to the manufacturer’s instructions. The RNA was

subsequently reverse-transcribed to cDNA by using a TOPscript™ reverse transcription (RT) kit (Enzynomics, Korea). Quantitative real-time PCR analysis was performed with an ABI7300 (Applied Biosystems, USA) using TOPreal™ qPCR 2X PreMIX (Enzynomics, Korea). The relative expression of the mRNAs was determined after normalization to cyclophilin A. Primer sets for real-time PCR: *Inhbe*, forward 5'-GCT AGC CAA GCA GCA AAT CC-3' and reverse 5'- GGA AGA TCC TCA GGT ACA GA-3'; *Atf4*, forward 5'-ATG ATG GCT TGG CCA GTG-3' and reverse 5'-CCA TTT TCT CCA ACA TCC AAT C; *Fgf21*, forward 5'-AGA TGG AGC TCT CTA TGG ATC G-3' and reverse 5'-GGG CTT CAG ACT GGT ACA CAT-3'; *Hsl*, forward 5'-GGG AGC ACT ACA AAC GCA AC-3' and reverse 5'-CAG AGA CGA CAG CAC CTC AA-3'; *Atgl*, forward 5'-CGC CTT GCT GAG AAT CAC CAT-3' and reverse 5'-AGT GAG TGG CTG GTG AAA GGT-3'; *Creb3l3*, forward 5'-CTG CCT CTC ACC AAG TAT G-3' and reverse 5'-AGT ACA CGC TGA CAT CCG-3'; *CycloA*, forward 5'-CAA GAC TGA ATG GCT GGA TG-3' and reverse 5'-TGG TGA TCT TCT TGC TGG TC-3'; *Srebp1c*, forward 5'-ATC GGC GCG GAA GCT GTC-3' and reverse 5'-ACT GTC TTG GTT GTT GAT-3'; *Fasn*, forward 5'-CCT GCT ATC ATC TGA CTT CCT CT-3' and reverse 5'-AGG GTG GTT GTT AGA AAG ATC AA-3'; *Acc1*, forward 5'-GCG GGA GGA GTT CCT AAT TC-3' and reverse 5'-TGT CCC AGA CGT AAG CCT TC-3'; *Ppara*, forward 5'-AAC TGG ATG ACA GTG ACA TTT CC-3' and reverse 5'-CCC TCC TGC AAC TTC TCA AT-3'; *Cpt1a*, forward 5'-TGG GCT ACT CAG AGG ATG G-3' and reverse 5'-AAG GTG TCA AAT GGG AAG G-3'; *Acadl*, forward 5'-TCT TTT CCT CGG AGC ATG ACA-3' and reverse 5'-GAC CTC TCT ACT CAC TTC TCC AG-3'; *Acox1*, forward 5'-TAA CTT CCT CAC TCG AAG CCA-3' and reverse 5'-AGT TCC ATG ACC CAT CTC TGT C-3'; *Mttp*, forward 5'-CTC TTG GCA GTG CTT TTT CTC T-3' and reverse 5'-GAG CTT GTA TAG CCG CTC ATT-3'; *ApoB*, forward 5'-TTG GCA AAC TGC ATA GCA TCC-3' and reverse 5'-TCA AAT TGG GAC TCT CCT TTA GC-3'; *Cd36*, forward 5'-ATT GGT CAA GCC AGC T-3' and reverse 5'-TGT AGG CTC ATC CAC TAC-3'.

Immunoblotting

Cells were lysed in cell lysis buffer (Cell Signaling Technology, USA) and subjected to immunoblotting. For tissue extraction, samples were pulverized in liquid nitrogen and homogenized in lysis buffer. Anti-phospho-Smad2 (Ser463/465), anti-Smad2, anti-p-HSL, anti-HSL, anti-p-PKA substrates, and β-Tubulin antibodies were purchased from

Cell Signaling Technology. Anti-Inhbe and anti-ALK7 antibodies were purchased from SantaCruz (USA). Proteins were detected by using ImageQuant LAS 4000 (GE Healthcare, UK).

Transient transfection and reporter gene assay

A 1.2 kb of mouse Inhbe promoter and a putative ATF4 response element mutant (-278 nt to -271 nt) in the *Inhbe* promoter were cloned into the luciferase reporter plasmid pGL4.2 (Promega, USA). AML12 cells were cultured in DMEM supplemented with 10% FBS and plated in a 24-well dish. Five hundred nanograms of each luciferase construct and 20 ng of Renilla luciferase pRL-TK plasmid (Promega, USA) with or without ATF4 overexpression vector (Addgene, USA) were transfected with Fugene HD (Life Technologies, USA). After 24 hr of incubation, lysates were prepared for luciferase assays using a Dual-Glo® luciferase assay kit (Promega, USA), and luciferase activity was measured using a Centro LB 960 Microplate Luminometer (Berthold Technologies, USA). Renilla luciferase activity was used as the control for normalization.

Chromatin immunoprecipitation (ChIP)

ChIP assays were performed with AML12 treated with 0.4 mM of palmitic acid or liver tissue from the mice fed high-fat diet for 4 weeks using a SimpleChIP® Enzymatic Chromatin IP Ki (Cell Signaling Technology, USA) according to the manufacturer's instructions. Antibodies against ATF4 (Cell Signaling Technology, USA) was used for chromatin immunoprecipitation. Precipitated DNA fragments were analyzed by quantitative real-time PCR using primers amplifying a 188 bp fragment encompassing the putative CRE sequence in the mouse *Inhbe* promoter: forward 5'-ACT GGA AGC ACT TCT TGG GG-3' and reverse 5'-CTT CCC ACC CTC TTT GCT GT-3'.

References for Supplementary Materials and Methods

1. Long, C. P. and Antoniewicz, M. R. High-resolution 13C metabolic flux analysis. *Nat. Protoc.* **14,** 2856–2877 (2019).
2. Wolfe, R. R. & Chinkes, D. L. *Isotope tracers in metabolic research: principles and practice of kinetic analysis*. 2nd edn (Wiley-Liss, 2005).

3. Kim, I. Y., Suh, S. H., Lee, I. K. and Wolfe, R. R. Applications of stable, nonradioactive isotope tracers in in vivo human metabolic research. *Exp. Mol. Med*. **48,** e203 (2016).
4. Turner, S. M. et al. Measurement of TG synthesis and turnover in vivo by 2H2O incorporation into the glycerol moiety and application of MIDA. *Am. J. Physiol. Endocrinol. Metab.* **285,** 790–803 (2003).
5. Jung, H. R., Turner, S. M., Neese, R. A., Young, S.G. and Hellerstein, M. K. Metabolic adaptations to dietary fat malabsorption in chylomicron-deficient mice. *Biochem. J*. **343**, 473–78 (1991).

Supplementary Figures

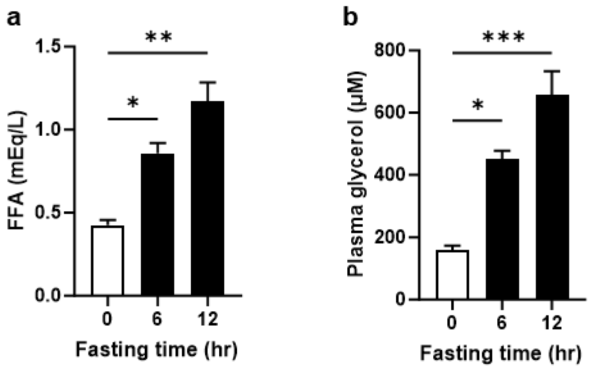

Supplementary Fig. 1. Plasma levels of free fatty acids (a) and free glycerol (b) as the fasting period increase.

The 12-weeks old male C57Bl/6J mice collected under ad-libitum, 6hr fasting, or 12hr fasting conditions (each group, n=3). Data are mean ± SEM. *, $p<0.05$; **, $p<0.01$; ***, $p<0.001$.

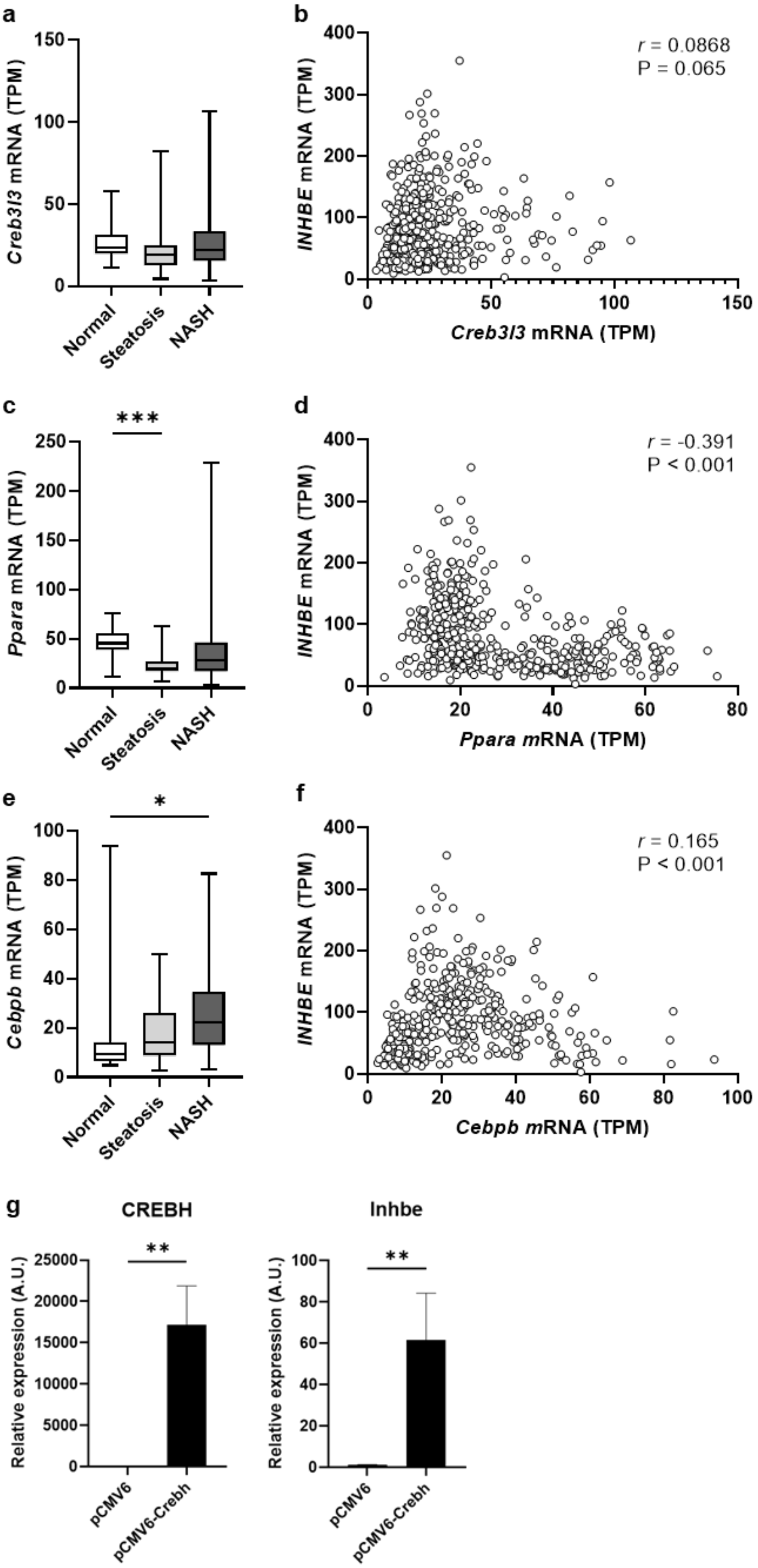
a
Creb3l3 mRNA (TPM)
Normal
Steatosis
NASH
b
r = 0.0868
P = 0.065
INHBE mRNA (TPM)
Creb3l3 mRNA (TPM)
c
***
Ppara mRNA (TPM)
Normal
Steatosis
NASH
d
r = -0.391
P < 0.001
INHBE mRNA (TPM)
Ppara mRNA (TPM)
e
*
Cebpb mRNA (TPM)
Normal
Steatosis
NASH
f
r = 0.165
P < 0.001
INHBE mRNA (TPM)
Cebpb mRNA (TPM)
g
CREBH
**
Relative expression (A.U.)
pCMV6
pCMV6-Crebh
Inhbe
**
Relative expression (A.U.)
pCMV6
pCMV6-Crebh

Supplementary Fig. 2. Gene expression correlation analysis in the public datasets of RNA sequencing from the liver of NAFLD patients.

a, c, e The TPM value of *Creb3l3*, *Ppara*, and *Cebpb* (Normal, n=39; Steatosis, n=179; NASH, n=260). **b, d, f** Gene expression correlation analysis of *INHBE* and each transcription factors in all samples from the dataset (Normal+Steatosis+NASH, n=454; r, Pearson correlation coefficient). TPM, transcripts per million. **g** The mRNA levels of *Creph and Inhbe* in AML12 hepatocytes under *Crebh* overexpression condition.

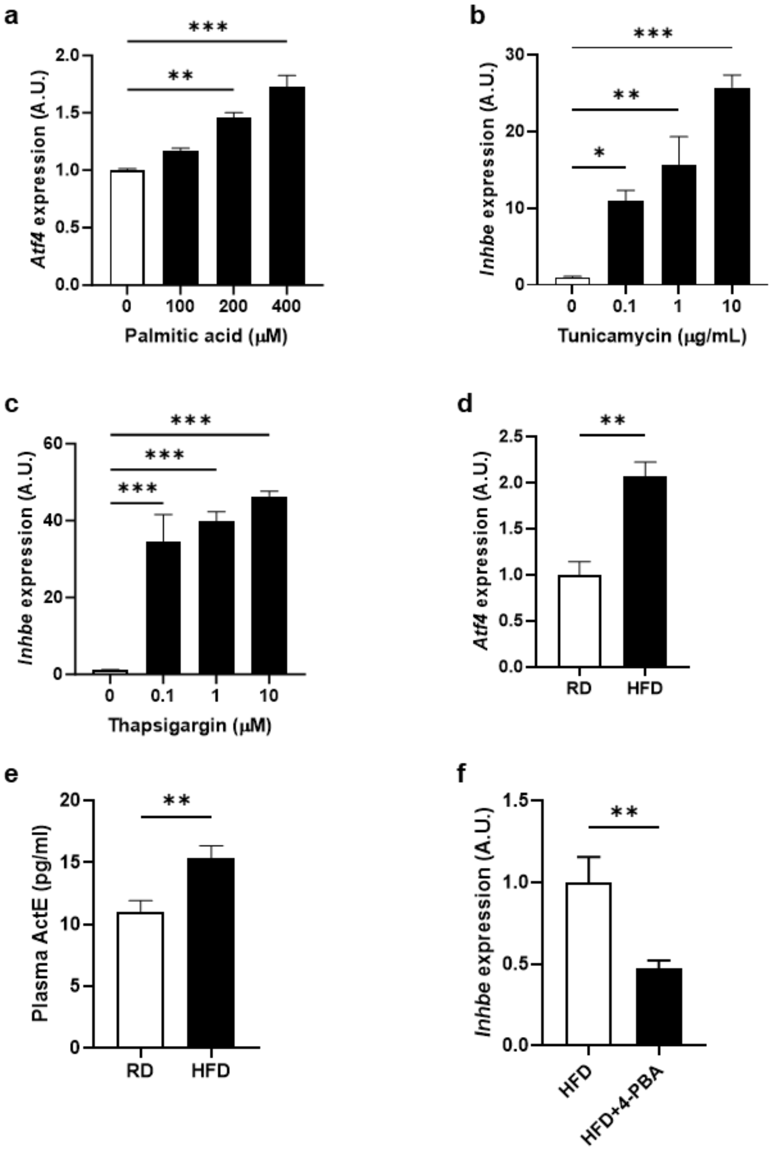

Supplementary Fig. 3. *Inhbe* expression is increased by hepatic ER stress.

a The mRNA levels of *Atf4* in AML12 hepatocytes were treated with palmitic acid for 6 hrs as indicated concentrations. **b and c** The mRNA level of *Inhbe* in AML12 cells treated with 0.4 mM of palmitate with or without Tunicamycin or Thapsigargin for 6 hrs as indicated concentrations. **d** The mRNA levels of *Atf4* in the livers of mice fed an HFD for 4 weeks. **e** The plasma level of Activin E fed a high-fat diet for 4 weeks (RD, n=6; HFD, n=6). **f** The mRNA level of *Inhbe* in the liver of mice fed a high-fat diet for 4 weeks treated with 4-phenyl butyric acid (4-PBA). Data are mean ± SEM. *, $p<0.05$; **, $p<0.01$; ***, $p<0.001$.

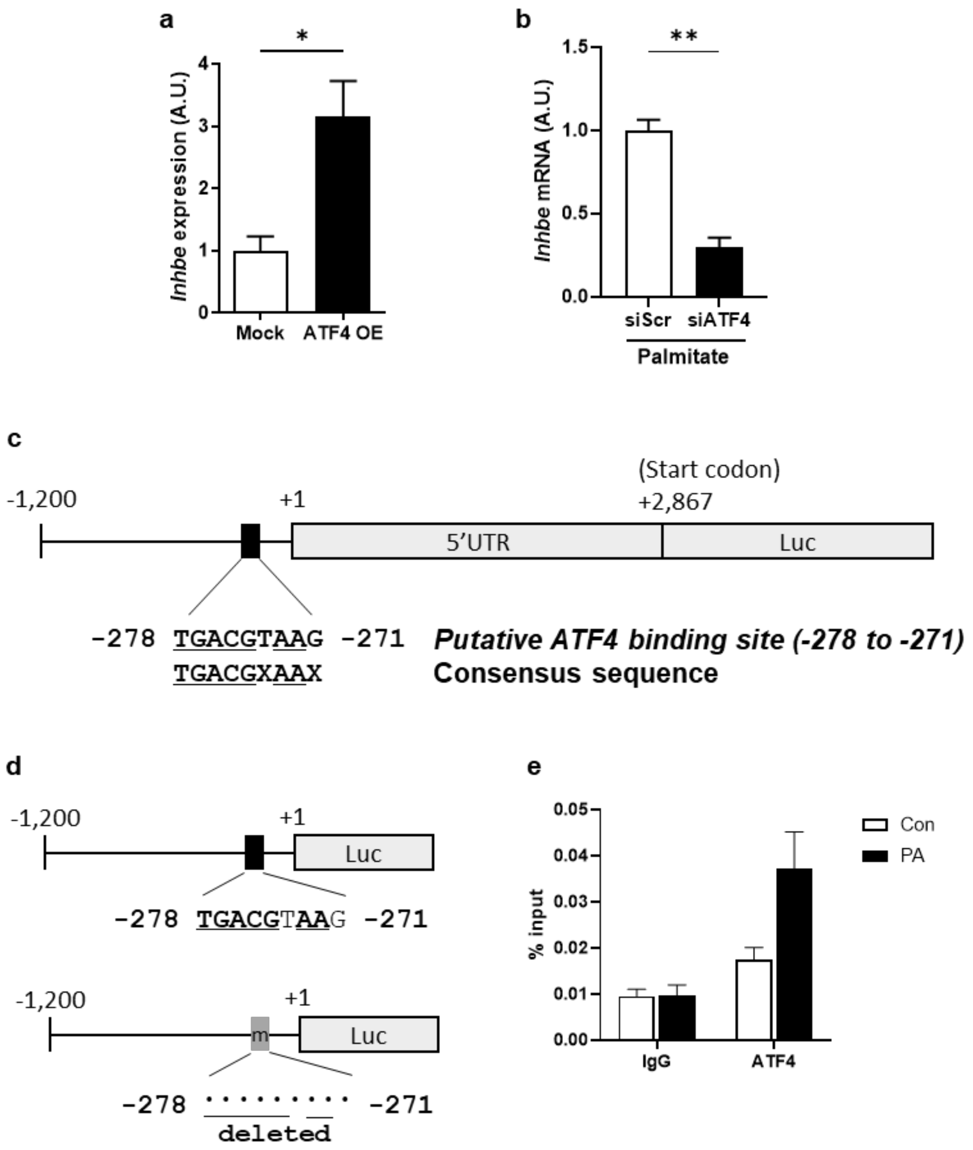

Supplementary Fig. 4. *Inhbe* expression is increased through ATF4 binding in the proximal promoter.

a The mRNA levels of *Inhbe* in AML12 hepatocytes under ATF4 overexpression condition. **b** The mRNA levels of *Inhbe* in AML12 hepatocytes under ATF4 knockdown condition. **c** A schematic representation of reporter vector including the promoter region (~1200 bp) of mouse *Inhbe*. **d** A schematic representation of reporter vector including the promoter region (~1200 bp) of mouse *Inhbe* or a putative ATF4 binding element-deleted promoter. **e** Chromatin immunoprecipitation (ChIP) assay with a specific anti-ATF4 antibody and primers for the putative ATF4-binding element of the Inhbe promoter in AML12 cells treated 0.4 mM of palmitic acid for 6 hrs. Data are mean ± SEM. *, p<0.05; **, p<0.01; ***, p<0.001.

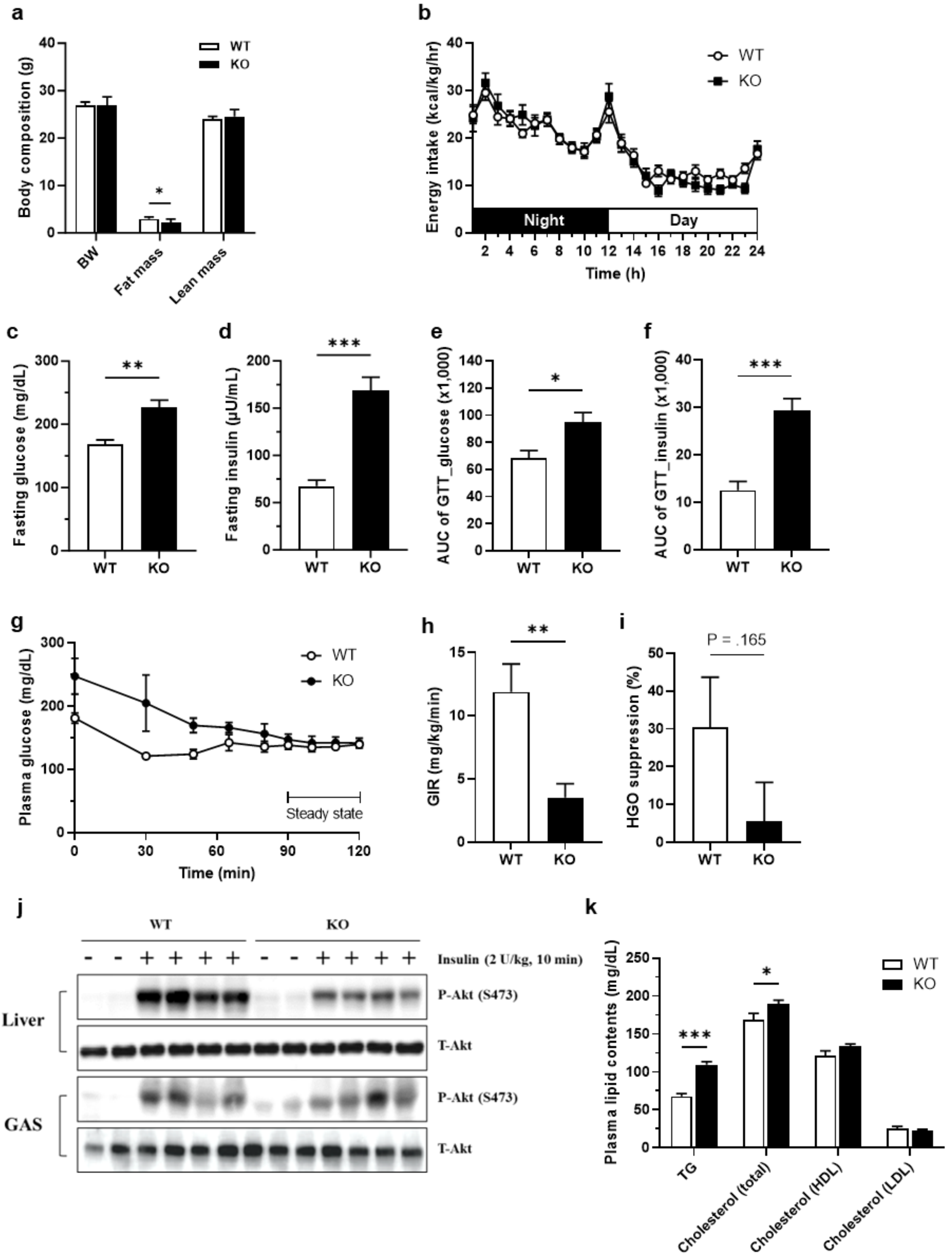

Supplementary Fig. 5. The *Inhbe* KO mouse has lean but lipodystrophic phenotypes.

a Body composition of the *Inhbe* WT and KO mice before HFD (WT, n=10; KO, n=10).

b Energy intake value of the WT and KO mice after HFD feed for 2 weeks (WT, n=13;

KO, n=7). **c** Fasting glucose levels of *Inhbe* WT and KO mice fed HFD for 4 weeks (WT, n=6; KO, n=8). **d** Fasting insulin level of *Inhbe* WT and KO mice fed HFD for 4 weeks (WT, n=6; KO, n=8). **e** Area under curve of plasma glucose level during GTT assay (WT, n=6; KO, n=8). **f** Area under curve of plasma insulin level during GTT assay (WT, n=6; KO, n=8). **g** Plasma glucose levels during hyperinsulinemic-euglycemic clamp. **h** Mean of glucose infusion rate (GIR). **i** Suppression of hepatic glucose output (HGO). **j** Western blot analysis for the phosphorylation of Akt (S473) in the liver and GAS tissues isolated from WT and KO mice injected insulin (2 U/Kg) for 10 min. **k** Plasma lipid profile of the WT and KO mice after HFD feed for 4 weeks (WT, n=9; KO, n=10). Data are mean ± SEM. *, $p<0.05$; **, $p<0.01$; ***, $p<0.001$.

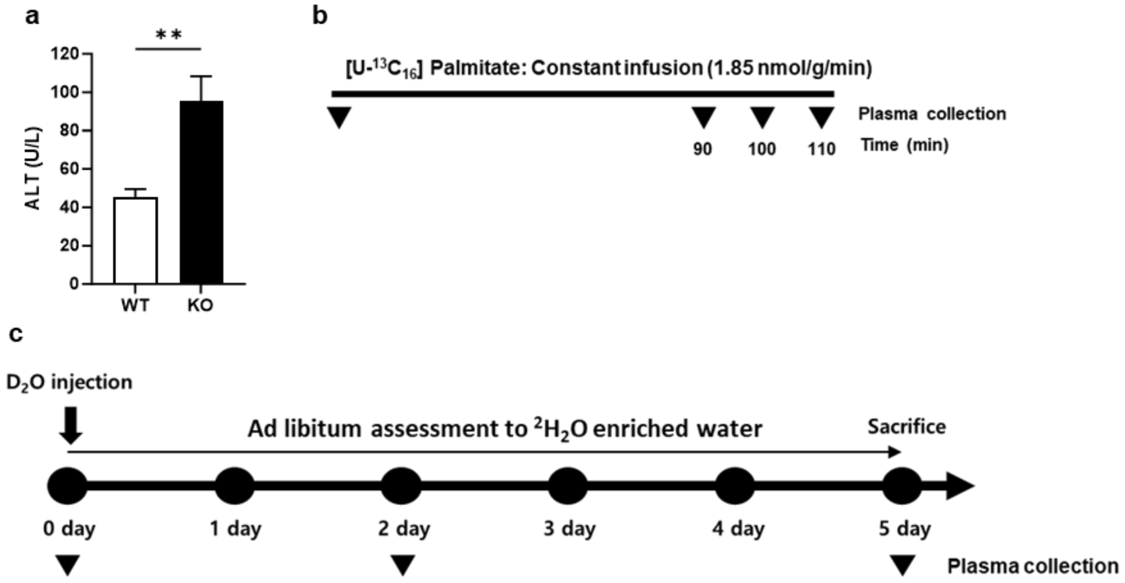

Supplementary Fig. 6. The *Inhbe* KO mouse has severe fatty liver phenotypes. **a** Plasma ALT levels of *Inhbe* WT and KO mice fed HFD for 4 weeks (WT, n=9; KO, n=10). **b** A protocol of systemic infusion of ^{13}C-labeled palmitate. **c** A protocol of D_2O labeling for measurement of hepatic TG synthesis rate and DNL rate. Data are mean ± SEM. *, p<0.05; **, p<0.01; ***, p<0.001.
